# FCRNet: Fast Fourier convolutional residual network for ventilator bearing fault diagnosis

**DOI:** 10.1371/journal.pone.0327342

**Published:** 2025-07-11

**Authors:** Yu Cao, Yongzhi Du, Likun Le, Xiaoxue Li, Yanfang Gao

**Affiliations:** 1 School of Mechanical and Electrical Engineering, China University of Mining and Technology, Beijing, China; 2 School of Mechanical and Transportation Engineering, Ordos Institute of Applied Technology, Inner Mongolia, China; The Hong Kong Polytechnic University, CHINA

## Abstract

This study presents FCRNet, a Fast Fourier Convolution Residual Network, tailored for fault diagnosis of mine ventilation bearings under complex operating conditions. By integrating residual learning with Fast Fourier Convolution (FFC), FCRNet employs a dual-branch architecture to effectively capture local spatial features and global frequency patterns. A Spectral Transformation (ST) module achieves unified processing of multi-scale spatial and frequency information by integrating local Fourier features (LFF), global fourier features (GFF), and local time-domain features (LF), overcoming the limitations of conventional convolutional approaches. The testing results on publicly available datasets and our self-built platform validate that the proposed method outperforms several existing fault diagnosis methods at various noise levels, providing strong support for the condition monitoring of mine ventilation.

## 1 Introduction

Mine ventilation is of utmost importance for coal mine safety [1]. In China, over 70% of casualties in mining fire and gas explosion incidents are caused by varying degrees of damage to ventilation facilities during disasters, resulting in localized or overall disruption of airflow, which leads to poisoning and asphyxiation among personnel [2]. Additionally, according to reports from the Mine Safety and Health Administration (MSHA), approximately 40% of mining accidents are attributed to poor air quality, highlighting the necessity of ventilation systems [3]. Therefore, mine ventilation fans are the primary power equipment for achieving economical and rational ventilation in mines [4], as well as for fire rescue ventilation, ensuring safe production [5]. As the “respiratory" system of the entire mine, the main ventilation fan operates continuously 24 hours a day, directly affecting the smoothness of airflow in the tunnels and the adequacy of air volume [6,7]. If ventilation is obstructed and the gas concentration rises to a certain level, it can easily lead to explosions and collapses, jeopardizing the safety of miners [8]. Given the critical nature of this safety equipment, any malfunction that causes a shutdown can result in significant economic losses or even catastrophic accidents [9,10]. Therefore, it is essential to conduct fault diagnosis on mine ventilation fans to enable early detection, prevention, and handling of fan faults, potential hazards, and adverse reactions, thereby avoiding major gas explosion incidents [11].

**Fig 1:**
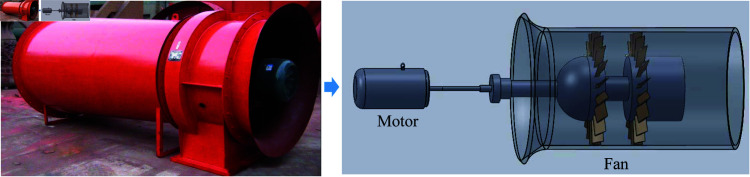
Schematic diagram of the ventilation fan and its main internal structure.

Mine ventilation fans typically use axial flow fans, which are directly connected to the motor, as shown in Fig 1. Rolling bearings (RB) are key components of the main fan [12], serving as the bridge between the rotating and stationary parts of the fan. Rolling bearings endure significant axial and radial loads, as well as dynamic loads caused by aerodynamic imbalances during fan operation. Therefore, failures in the rolling bearings can lead to damage to the main fan, and their condition directly affects the operation of the equipment, potentially resulting in prolonged production interruptions. Thus, effective fault diagnosis of mine ventilation fan bearings is crucial for ensuring the safe and reliable operation of equipment, identifying problems early, reducing risks, and ensuring operational continuity [13].

Traditionally, bearing fault diagnosis relies on signal processing methods (such as Fourier transform [14] and wavelet analysis [15]) and statistical feature extraction to detect anomalies in vibration data [16]. However, the complex mining environment, characterized by harsh operating conditions and high levels of noise, renders these methods less applicable in terms of noise resistance, stability, and accuracy. With the development of big data, the Internet of Things, and computer technology, particularly the emergence of deep learning techniques, the issue of bearing fault diagnosis has reached a new peak in research [17–19]. In particular, powerful networks such as convolutional neural networks (CNN) [20–24] can automatically extract features from raw vibration signals, significantly surpassing traditional manual feature extraction methods in capturing spatial patterns. Jia *et al*. [25] proposed a network called the Gramian Time-Frequency Enhancement Network (GTFE-Net) for bearing fault diagnosis, which can reduce unwanted noise in vibration signals and achieve significant improvements in classification performance. Li *et al*. [26] proposed an enhanced fault detection framework based on CNN, long short-term memory network (LSTM) and Kolmogorov-Arnold network (KAN), which improves the model’s robustness and accuracy under conditions of data scarcity and complex spatiotemporal relationships. Zhao *et al*. [27] proposed a normalized convolutional neural network that takes into account data imbalance and variable working conditions for diagnosing different severity levels and directions of faults. Wang *et al*. [28] proposed a fault diagnosis method based on optimal synchronous wavelet transform (SWD) and one-dimensional convolutional neural networks (1D-CNN), achieving good generalization results in multi-sensor data fusion. Chen *et al*. [29] proposed a particle swarm optimization (PSO) algorithm to optimize the CNN architecture, achieving higher accuracy than other state-of-the-art methods. Huang *et al*. [30] proposed a new method called Enhanced Transformer with Asymmetric Loss Function (ETALF) to address the issue of intelligent fault diagnosis with noisy labels under varying working conditions. Wang *et al*. [31] proposed a novel attention-guided joint learning convolutional neural network (JL-CNN) for fault diagnosis, which exhibits excellent fault diagnosis capabilities and signal denoising performance, particularly under strong and unknown noise conditions.

In practical industrial applications, to customize deeper networks based on large volumes of data, Residual Networks (ResNet) [32] have introduced skip connections to enhance gradient flow in deep architectures. Peng *et al*. [33] proposed a deeper one-dimensional convolutional neural network (Der-1DCNN). This framework incorporates the concept of residual learning, enabling it to effectively learn high-level and abstract features while alleviating the challenges of training and the performance degradation associated with deeper networks. Long *et al*. [34] first developed a method to convert time-domain fault signals into RGB image format, and then proposed a novel TCNN (ResNet-50) architecture for bearing fault diagnosis, which outperformed other deep learning models and traditional methods. Liang *et al*. [35] proposed a novel fault diagnosis method for rolling bearings based on wavelet transform (WT) and an improved residual neural network (IResNet) to address the issue of fault diagnosis with label noise.

However, The complex working environment of mine ventilation systems introduces high levels of background noise and multi-source interference, including vibration transmission from adjacent equipment, airflow disturbances, and environmental factors such as mine blasting [36,37], hampering effective feature extraction by time-domain convolutional networks. Furthermore, mine ventilators operate under variable speed conditions and load fluctuations, causing dynamic changes in fault characteristic frequencies that traditional convolutional architectures struggle to capture [38]. The common solution is to stack more convolutional modules. Although architectures like ResNet improve gradient propagation through residual connections, their core time-domain convolution operations have limited capability to capture frequency-domain features crucial for bearing fault detection [39]. To address these challenges, this paper introduces Fast Fourier Convolution (FFC) technology, an innovative approach that enables multi-scale representation of vibration signals through simultaneous feature extraction in both frequency and spatial domains [40]. Unlike traditional convolution, FFC effectively separates high-frequency and low-frequency signal components, providing a significant advantage in processing bearing fault signals with pronounced frequency-domain characteristics.

Therefore, by combining FFC with ResNet, it is possible to enhance feature representation in both spatial and frequency domains, providing a pathway to overcome the limitations of existing methods and improve the diagnostic accuracy of mine ventilator bearings under challenging operating conditions.

The main contributions of this article are as follows.

(1) This paper proposes FCRNet, a Fast Fourier Convolution Residual Network, tailored for fault diagnosis of mine ventilation fan bearings.
(2) A Spectral Transform (ST) module that processes multi-scale spatial and frequency information, overcoming the local focus of traditional CNNs.
(3) An optimized fusion of residual learning and FFC, ensuring efficient feature propagation and adaptability to complex operating conditions.

The rest of this article is organized as follows. Sect 2 introduces the basic forms of residual structures and fast Fourier convolution. Sect 3 presents the proposed FCRNet and the fault diagnosis framework for ventilator bearings. Sect 4 provides a comprehensive validation of the proposed method. Finally, Sect 5 concludes this article.

## 2 Overview of ResNet and Fast Fourier convolution

### 2.1 Residual neural network

To address the significant degradation problem faced by deep neural networks during training, the residual learning framework proposes a new approach for optimizing deep neural networks. The core idea is to learn the residual mapping *F*(*x*) instead of directly learning the desired feature mapping *H*(*x*). This can be expressed as:

F(x)=H(x)−x (1)

Thus, the original feature mapping can be rewritten as:

H(x)=F(x)+x (2)

where *x* is the input feature, *F*(*x*) is the residual mapping, and *H*(*x*) is the desired feature mapping.

**Fig 2:**
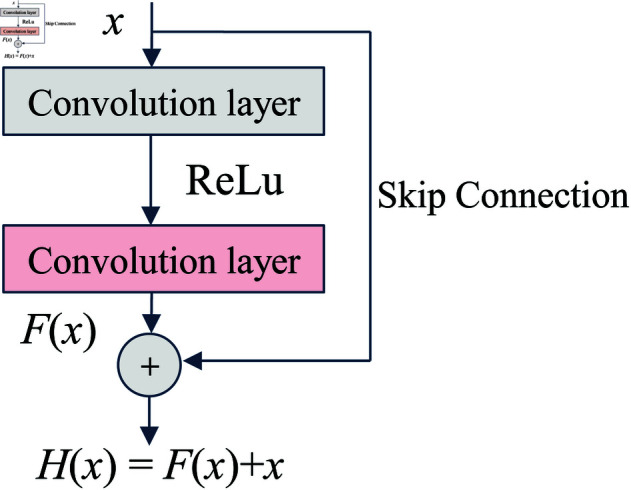
The basic structure of residual network.

The fundamental building block of a residual neural network is the residual block. A typical residual block consists of two paths: the main path (residual branch) and the shortcut connection, as shown in Fig 2. It utilizes skip connection (shortcut connection) to combine shallow features with deep features obtained through multiple convolution operations. With this design, instead of directly learning the original features, the network now only needs to learn the residual between the input and output. When the *F*(*x*) is 0, the network layer does not perform a skip connection, but it still receives the input *x*, allowing the network to extract certain features. However, in practice, the residual is unlikely to be 0, which enables the network layer to learn new features from the skip connection in addition to those from the input *x*. The combination of these two aspects results in improved performance.

The mathematical expression for a basic residual block is:

$$y=F(x,\{W_{i}\})+x$$ (3)

where *F*(*x*,{*W*_*i*_}) represents the residual mapping and {*W*_*i*_} is the set of weight parameters. Specifically, the basic residual block includes the following components:

1) Main Path: 3×3 convolution layer - Batch Normalization (BN) - ReLU activation function - 3×3 convolution layer - Batch Normalization
2) Shortcut Connection:Direct connection or 1×1 convolution (for dimension matching)

An important characteristic of residual networks is their excellent gradient propagation properties. For any deep layer *L*, the gradient of the loss function ε can be expressed using the chain rule as:

$$\frac{\partial \varepsilon }{\partial x_{l}}=\frac{\partial \varepsilon }{\partial x_{L}}\cdot \frac{\partial x_{L}}{\partial x_{l}}=\frac{\partial \varepsilon }{\partial x_{L}}\cdot \left(1+\frac{\partial }{\partial x_{l}}\sum_{i=l}^{L-1}F(x_{i},W_{i})\right)$$ (4)

Eq (4) indicates that the gradient contains at least one identity term, effectively preventing the vanishing gradient problem. Even if the gradient of the residual term approaches zero, the network can still perform effective parameter updates through the shortcut connections.

### 2.2 Fast Fourier convolution

**Fig 3:**
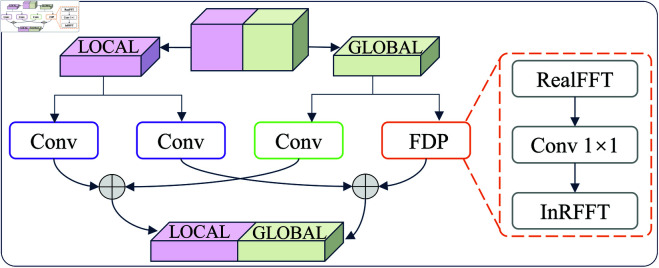
The structure of the FFC.

Fast Fourier Convolution (FFC) is an innovative neural network that effectively addresses the limitations of local perception in traditional convolutional neural networks by integrating frequency domain processing (FDP) with spatial domain operations, as illustrated in the Fig 3. The core idea is to divide the input feature channels into two parallel branches: the local branch retains traditional convolution operations to capture detailed features, while the global branch employs Real-valued Fast Fourier Transform (RFFT) for feature extraction in the frequency domain, thereby obtaining global contextual information. Compared to standard FFT, RFFT utilizes only half of the spectrum to perform computations, significantly enhancing computational efficiency. This dual-branch architecture not only reduces computational complexity but also enables long-range dependency modeling across spatial locations while maintaining the ability to extract local features, making it particularly suitable for tasks that require simultaneous attention to both local details and global features. FDP of FFC makes following steps:

1) Apply the RFFT on the frequency dimension of the input feature map, and concatenate the real and imaginary parts of the spectrum along the channel dimension:
  $${\mathbb{R}}^{H\times W\times C}\to {\mathbb{C}}^{H\times \frac{W}{2}\times C}\to {\mathbb{R}}^{H\times \frac{W}{2}\times 2C}$$ (5)
  The input feature map, sized ${\mathbb{R}}^{H\times W\;\times \;C}$, undergoes RFFT along the width dimension (*W*) to convert it from the spatial to the frequency domain. Leveraging the symmetry of real-valued inputs, RFFT computes only half the frequency spectrum ($\frac{W}{2}$), yielding a complex spectrum of size $H\;\times \;\frac{W}{2}\;\times \;C$ in ℂ. To enable subsequent convolution, the real and imaginary parts of each channel’s spectrum ($H\;\times \;\frac{W}{2}\;\times \;1$) are separated and concatenated along the channel dimension, doubling the channels from *C* to 2*C* and forming a real-valued feature map of size $H\;\times \;\frac{W}{2}\;\times \;2C$. This transformation captures global frequency information, including low-frequency patterns and high-frequency details, preparing it for standard convolutional processing.
2) Apply a convolutional block (with a 1×1 kernel) in the frequency domain:
  $${\mathbb{R}}^{H\times \frac{W}{2}\times 2C}\to {\mathbb{R}}^{H\times \frac{W}{2}\times 2C}$$ (6)
  A 1×1 convolutional kernel processes the frequency-domain feature map from Step 1, performing linear combinations and non-linear transformations across channels without altering the spatial dimensions ($H\;\times \;\frac{W}{2}$). The output retains the size $H\;\times \;\frac{W}{2}\;\times \;2C$. In the frequency domain, this operation weights and fuses frequency components, enhancing or suppressing specific features (e.g., fault-related frequency patterns), enabling the model to learn complex frequency patterns efficiently due to the low computational cost of 1×1 convolutions.
3) Apply the inverse RFFT (InRFFT) to restore the spatial structure:
  $${\mathbb{R}}^{H\times \frac{W}{2}\times 2C}\to {\mathbb{C}}^{H\times \frac{W}{2}\times C}\to {\mathbb{R}}^{H\times W\times C}$$ (7)
  The frequency-domain feature map from Step 2, with 2*C* channels, is reorganized by pairing real and imaginary parts to form a complex spectrum of size $H\times \frac{W}{2}\times C$ in ℂ. An inverse Real-valued Fast Fourier Transform (IRFFT) is then applied, leveraging the symmetry of RFFT to reconstruct the full width *W*, yielding a spatial-domain feature map of size H×W×C in ℝ. This step converts frequency-processed features back to the spatial domain, integrating global frequency information while preserving spatial structure for compatibility with subsequent convolutional layers and tasks like classification or segmentation.

These three steps collectively form the global branch processing flow of the FFC. By operating in the frequency domain, FFC can capture long-range dependencies and global contextual information (thanks to the global nature of the Fourier transform), while efficiently processing frequency components using 1×1 convolutions. When combined with the local branch of FFC (traditional convolution), this dual-branch architecture models global features while preserving local details, making it particularly suitable for bearing fault detection, which requires simultaneous attention to both local and global information (with high-frequency components corresponding to fault impacts and low-frequency components capturing background noise).

## 3 Proposed method

### 3.1 Design of FCRNet block

**Fig 4:**
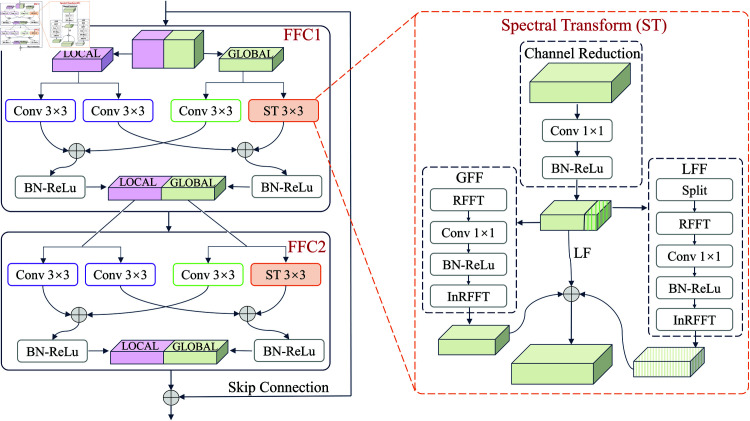
The structure of the proposed FCRNet block.

Based on the theoretical foundations of ResNet and Fast Fourier Convolution discussed above, we propose a novel network architecture called FCRNet (Fast Fourier Convolution Residual Network) for **rotating machinery** fault diagnosis. The block of the proposed FCRNet is shown in the Fig 4. Each residual block consists of two Fast Fourier Convolution units (FFC1 and FFC2), with each unit processing different feature information through local and global paths, respectively.

In the local path, standard 3×3 spatial convolution (Conv 3×3) is used to extract local spatial features, which are then enhanced for nonlinear representation through Batch Normalization (BN) and the ReLU activation function.

The global path is achieved through Spectral Transform (ST 3×3), where the input is first mapped from the spatial domain to the frequency domain, allowing for convolution operations on global features, and then the inverse spectral transformation is applied to restore the data to the spatial domain. This design effectively captures global feature frequency patterns in the frequency domain while preserving the integrity of the spatial domain information.

The outputs from the two paths are fused using an addition operation and then processed through BN-ReLU operations before being combined into a joint representation of local and global features, which serves as the output of the residual block. By introducing residual connections, the model effectively alleviates the vanishing gradient problem and enables efficient feature propagation in deep networks.

### 3.2 Spectral Transform (ST)

The ST network is designed to efficiently process both spatial and frequency domain features through a multi-branch architecture, as shown in Fig 4. The module first reduces the channel dimension for computational efficiency, then processes features through three parallel paths (local spatial, global frequency, and local frequency), and finally restores the channel dimension while preserving spatial information. The detailed mathematical formulation is presented below.

Let $X\in {\mathbb{R}}^{B\times C\times H\times W}$ denote the input feature map, where *B* represents the batch size, *C* denotes the number of channels, and *H*, *W* are the spatial dimensions (height and width) respectively.

0) Channel Reduction: The input features are first processed through a dimensionality reduction operation:

$$X_{reduced}=\text{ReLU}(\text{BN}({\text{Conv}}_{1\times 1}(X)))\in {\mathbb{R}}^{B\times \frac{C}{2}\times H\times W}$$ (8)

where ${\text{Conv}}_{1\times 1}$ denotes a 1×1 convolution operation, BN represents batch normalization, and ReLU is the rectified linear unit activation function.

1) Local Feature Extraction (LF): The reduced features directly serve as local spatial features:

$$X_{local}=X_{reduced}\in {\mathbb{R}}^{B\times \frac{C}{2}\times H\times W}$$ (9)

2) Global Frequency Domain Feature Extraction (GFF): The Fourier Unit processes the reduced features in the frequency domain:

$$F=ℱ(X_{reduced})\;F_{real}=\text{Real}(F)\in {\mathbb{R}}^{B\times \frac{C}{2}\times H\times \frac{W}{2}+1\times 2}\;F_{conv}=\text{ReLU}(\text{BN}({\text{Conv}}_{1\times 1}(F_{real})))\;X_{global}=\text{Real}({ℱ}^{-1}(F_{conv}))\in {\mathbb{R}}^{B\times \frac{C}{2}\times H\times W}$$ (10)

where ℱ and ${ℱ}^{-1}$ denote the 2D Fourier transform and its inverse transform respectively. The Real(·) operation extracts the real component of complex numbers.

3) Local Frequency Domain Feature Extraction (LFF): To capture local frequency patterns, the features are processed in patches:

$$X_{split}=\text{Split}(X_{reduced},H/2,W/2)\;F_{local}=ℱ(X_{split})\;F_{local\_conv}=\text{ReLU}(\text{BN}({\text{Conv}}_{1\times 1}(F_{local})))\;X_{local\_f}=\text{Real}({ℱ}^{-1}(F_{local\_conv}))\cdot \text{Repeat}(2,2)\in {\mathbb{R}}^{B\times \frac{C}{2}\times H\times W}$$ (11)

where Split(·) divides the feature map into 2×2 patches, and Repeat(2,2) repeats the features to restore the original spatial dimensions.

4) Feature Fusion and Channel Restoration: The three parallel paths are combined and processed to restore the original channel dimension:

$$X_{fused}=X_{local}+X_{global}+X_{local\_f}\in {\mathbb{R}}^{B\times \frac{C}{2}\times H\times W}$$ (12)

$$Y={\text{Conv}}_{1\times 1}(X_{fused})\in {\mathbb{R}}^{B\times C\times H\times W}$$ (13)

where *Y* represents the final output feature map with restored channel dimension.

The ST module effectively combines multi-scale spatial and frequency information through its parallel processing architecture. The channel reduction and restoration operations ensure computational efficiency while maintaining the network’s representation capacity. This design is particularly effective for processing vibration signals, where both spatial patterns and frequency characteristics are crucial for fault diagnosis.

### 3.3 Ventilation bearing fault diagnosis framework based on FCRNet

**Fig 5:**
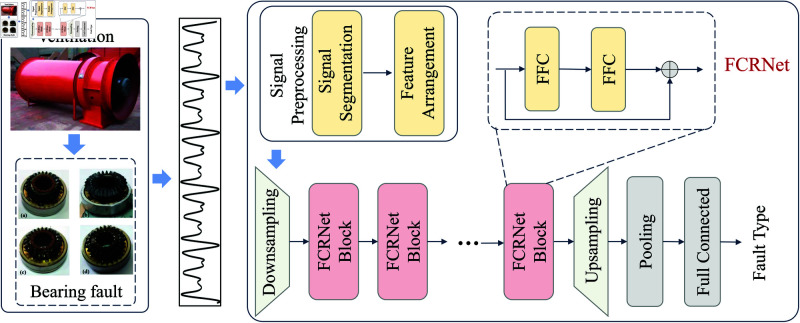
Fault diagnosis framework for ventilator bearings based on FCRNet.

The proposed axial Fan bearing fault diagnosis framework based on FCRNet is shown in Fig 5. This framework aims to accurately diagnose bearing fault types by leveraging the multi-scale modeling capabilities of signal features and the efficient capture of frequency domain characteristics. Specifically, the workflow of the framework can be divided into the following stages:

1) Signal Preprocessing: The proposed FCRNet architecture requires a 2D input tensor $X\in {\mathbb{R}}^{B\times C\times H\times W}$. However, the raw vibration signals collected from axial fan are typically one-dimensional time series data $S\in {\mathbb{R}}^{B\times C\times L}$, where *L* represents the length of the signal sequence. Therefore, a preprocessing step is necessary to transform the 1D signals into 2D feature maps. The transformation process can be formulated as follows:
  a) Signal Segmentation: First, the original signal sequence is divided into segments of length *W*:
  $$S_{seg}=\text{Reshape}(S,(B,C,H,W))$$ (14)
  where H=⌈L/W⌉ represents the number of segments, and zero-padding is applied if necessary to ensure *L* is divisible by *W*.
  b) Feature Arrangement: The segmented signals are then arranged into a 2D matrix form:
  $$X=S_{seg}\in {\mathbb{R}}^{B\times C\times H\times W}$$ (15)
  This transformation preserves both the temporal correlations within each segment (along the *W* dimension) and the long-term dependencies between segments (along the *H* dimension). The resulting 2D feature maps *X* can be effectively processed by the subsequent FCRNet while maintaining the essential characteristics of the original vibration signals.
2) Downsampling and FCRNet Block Processing: Firstly, the input signals are first processed through a downsampling module to reduce dimensionality, extracting initial low-level features while lowering computational complexity and retaining critical fault information. Then the core processing module consists of multiple cascaded FCRNet blocks. Each FCRNet block is composed of several stacked Fast Fourier Convolution units (FFC), divided into local and global paths to extract features in both the spatial and frequency domains. The local path captures spatial structural characteristics using traditional convolution operations, while the global path processes features through Spectral Transformation (ST) to capture global feature frequency patterns in the frequency domain. Residual connections within the FCRNet blocks facilitate efficient feature propagation and enhance the network’s convergence speed and generalization capability.
3) Upsampling and Classification: After multi-scale feature extraction, the framework employs an upsampling module to restore feature resolution, followed by pooling layers and fully connected layers for final fault type classification. The fully connected layer outputs confidence scores for each fault type, enabling accurate diagnosis.

## 4 Experiments and discussion

### 4.1 Experimental setup and data processing

#### 4.1.1 Experimental rig.

**Fig 6:**
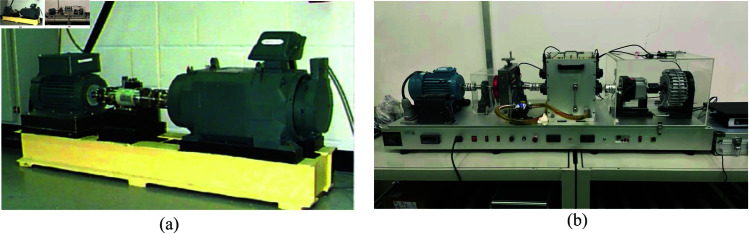
Test rig. (a) Public fataset CWRU, (b) Our self-constructed dataset SBTP.

To validate the effectiveness of the proposed method, tests were conducted on the publicly available CWRU bearing dataset and on our self-built bearing testing platform (SBTP). The experimental setup is illustrated in Fig 6.

The CWRU dataset encompasses ten different bearing conditions. The fault conditions were consist of three types of bearing faults (Inner race fault, Outer race fault, Rolling element (ball) fault). For each fault type, three different fault diameters were created: 0.007 inches (0.178 mm), 0.014 inches (0.356 mm), and 0.021 inches (0.533 mm).

The SBTP dataset includes a total of seven types: normal, cage fault, rolling element fault, inner race fault, outer race fault, inner race and outer race combined fault, and inner race and outer race with rolling element combined fault.

**Fig 7:**
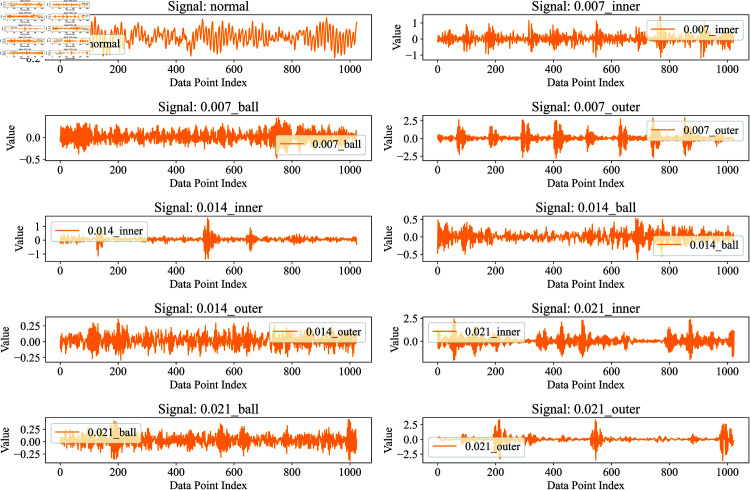
The CWRU dataset contains the first 1024 signal points for each different fault type.

**Fig 8:**
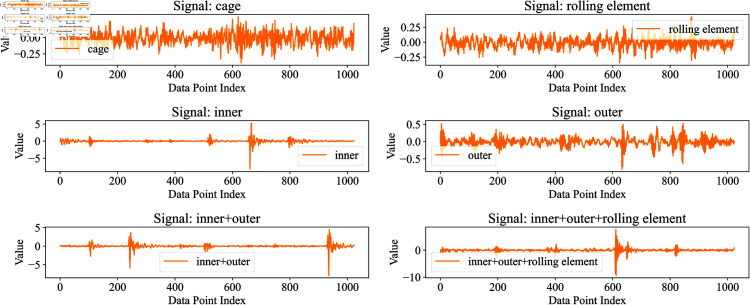
The SBTP dataset contains the first 1024 signal points for each different fault type. (The normal type has been excluded.)

Representative vibration signals from different bearing conditions are illustrated in Figs 7 and 8. The temporal waveforms demonstrate distinct characteristics across various fault types and severity levels, highlighting the discriminative patterns that can be leveraged for fault diagnosis.

#### 4.1.2 Data processing.

**Fig 9:**
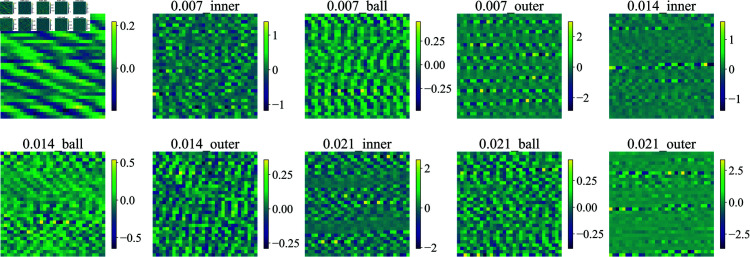
Feature map results of CWRU dateset.

**Fig 10:**
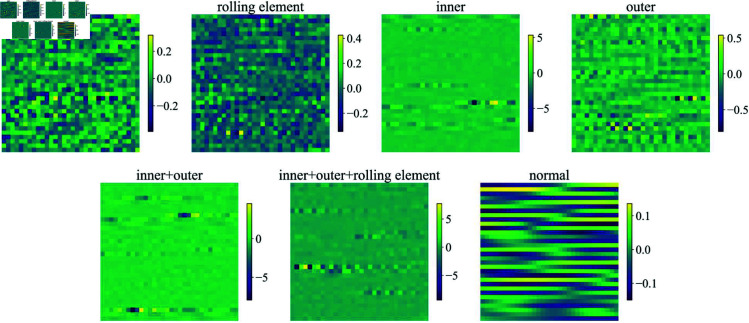
Feature map results of SBTP dateset.

According to Formula 15, it can be known that the choice of segment length *W* is an important hyperparameter that affects the network’s performance. A larger *W* allows the network to capture longer temporal patterns within each segment, while a smaller *W* provides finer granularity in the temporal dimension. In our implementation, we set *W* = 32, which provides a good balance between temporal resolution and computational efficiency. The Figs 9 and 10 show the feature map results of different fault type signals when *W* = *H* = 32. These feature maps will serve as the input of FCRNet.

### 4.2 Fault diagnosis results of FCRNet

**Experimental and tool configuration:** To evaluate the effectiveness of the proposed method in fault diagnosis tasks, the dataset was partitioned into training, validation, and testing sets with a ratio of 7:2:1. Comprehensive performance evaluations were conducted across all three sets. All experiments were conducted on a workstation equipped with an Intel Xeon E5-2698 CPU, 64 GB of RAM, and an NVIDIA RTX 3090 GPU. The FCRNet model was implemented using PyTorch 2.0 and CUDA 11.7 on an Ubuntu 22.04 system. The FCRNet network structure and its main parameters are shown in Table 1. Each residual block consists of a combination of two FFCs, BatchNorm, ReLU activation functions, and skip connections. The total number of parameters in the network is 1.24 million, which provides a richer feature representation while maintaining lower computational complexity compared to standard ResNet structures of equivalent depth. Additionally, in the experiments, data preprocessing and augmentation were implemented using NumPy (version 1.20.3) and SciPy (version 1.10.1). Model performance evaluation was conducted using scikit-learn (version 1.3.0) to calculate metrics such as accuracy and F1 score, while Matplotlib (version 3.7.2) and Seaborn (version 0.13.2) were used for visualizing the comparison results. Dataset management and preprocessing were handled with Pandas (version 2.0.3) to ensure compatibility between the data format and the model.

**Table 1:** FCRNet network structure and parameter configuration.

Layer	Output Size	Convolution Kernel/Operation	Number of Channels	Repetitions
Input Layer	32×32	-	1	-
Convolution Layer	16×16	3×3, stride=2	16	1
Residual Block Group 1	16×16	3×3 FFC	16	2
Residual Block Group 2	8×8	3×3 FFC	32	2
Residual Block Group 3	4×4	3×3 FFC	64	2
Residual Block Group 4	2×2	3×3 FFC	128	2
Average Pooling	1×1	Adaptive Pooling	128	1
Fully Connected Layer	4	-	4	1

**Fig 11:**
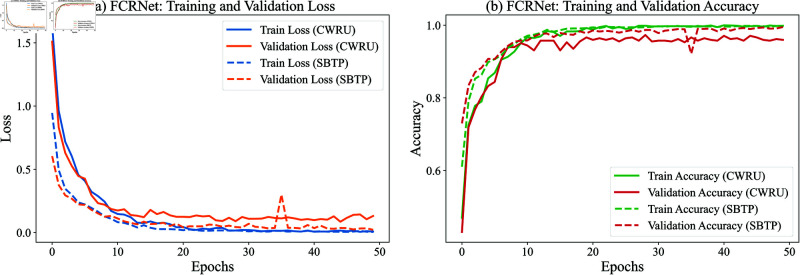
Loss and accuracy results of the training process.

**Training process analysis**: Fig 11 illustrates the evolution of loss and accuracy metrics during the training process. The loss curves (Fig 11(a)) demonstrate rapid convergence for both training and validation sets, indicating efficient optimization of the model parameters. The accuracy curves (Fig 11(b)) exhibit consistent improvement, ultimately approaching 1.0 for both sets, which suggests robust generalization capability without overfitting.

**Fig 12:**
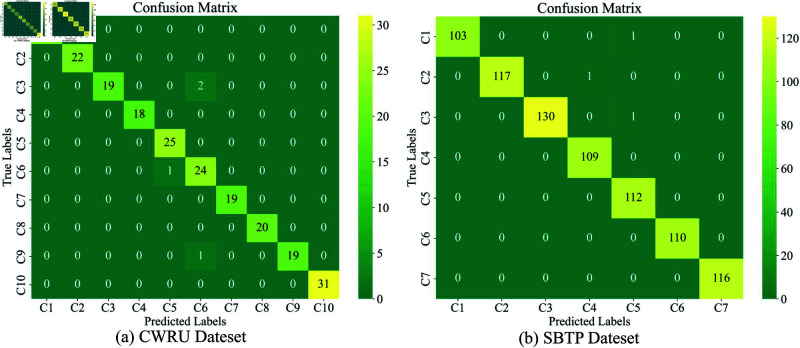
Confusion matrix in the test dataset, (a) CWRU dateset, (b) SBTP dateset.

**Fig 13:**
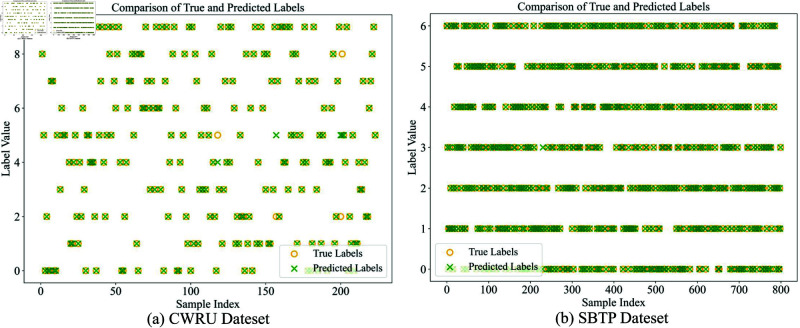
Comparison of true and predicted labels, (a) CWRU dateset, (b) SBTP dateset.

**Classification performance evaluation**: The confusion matrix for the test set is presented in Fig 12, revealing excellent classification performance across most fault categories, demonstrating the model’s strong discriminative capability across different fault types. Fig 13 presents a comparison between true and predicted labels, showing remarkable consistency and further validating the reliability of the classification results.

**Detailed performance metrics:** Tables 2 and 3 presents a comprehensive analysis of performance metrics including Precision, Recall, F1-Score for each fault category. For CWRU, precision, recall, and F1-scores reach 100% for eight categories, with slight drops in Class 3 (recall: 90.48%) and Class 6 (precision: 88.89%). Macro- and weighted averaged F1-scores of 98.28% and 98.22% indicate strong performance despite class variations. For SBTP (7 classes), metrics exceed 98% across all categories, with macro- and weighted averaged F1-scores of 99.62% and 99.63%. This demonstrates the superiority of FCR in practical diagnostics.

**Table 2:** Different testing metrics for each category of the CWRU dataset.

Class	Precision × 100%	Recall × 100%	F1-Score × 100%
1	1.0000	1.0000	1.0000
2	1.0000	1.0000	1.0000
3	1.0000	0.9048	0.9500
4	1.0000	1.0000	1.0000
5	0.9615	1.0000	0.9804
6	0.8889	0.9600	0.9231
7	1.0000	1.0000	1.0000
8	1.0000	1.0000	1.0000
9	1.0000	0.9500	0.9744
10	1.0000	1.0000	1.0000
macro-averaged	0.9850	0.9815	0.9828
weighted-averaged	0.9833	0.9821	0.9822

**Table 3:** Different testing metrics for each category of the SBTP dataset.

Class	Precision × 100%	Recall × 100%	F1-Score × 100%
1	1.0000	0.9904	0.9952
2	1.0000	0.9915	0.9957
3	1.0000	0.9924	0.9962
4	0.9909	1.0000	0.9954
5	0.9825	1.0000	0.9912
6	1.0000	1.0000	1.0000
7	1.0000	1.0000	1.0000
macro-averaged	0.9962	0.9963	0.9962
weighted-averaged	0.9963	0.9962	0.9963

**Fig 14:**
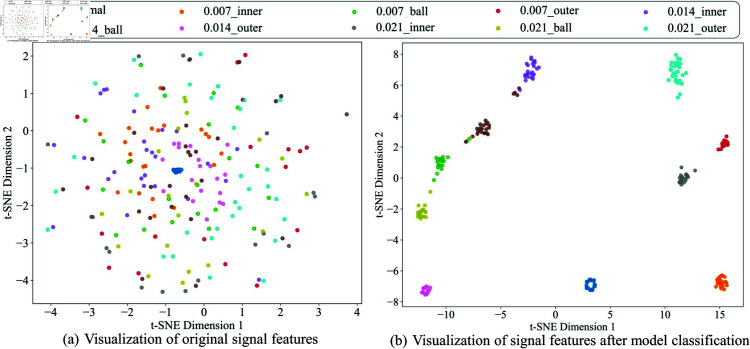
Feature visualization results of CWRU.

**Fig 15:**
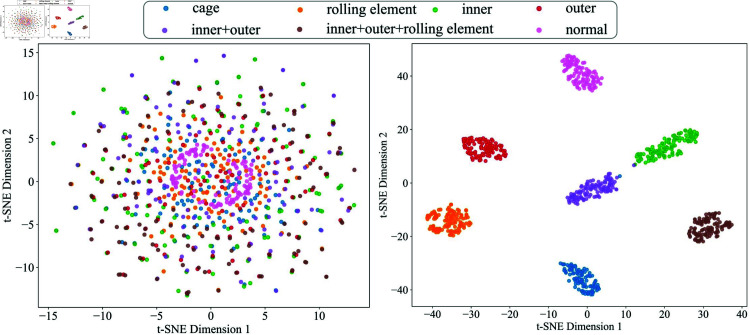
Feature visualization results of SBTP.

**Feature visualization analysis**: Figs 14 and 15 illustrate the effectiveness of the feature representations through t-SNE dimensionality reduction. In Figs 14(a) and 15(a), the original signal features show a more scattered distribution with significant overlap between fault categories. However, in Figs 14(b) and 15(b), the features after model classification form distinct and well-separated clusters, corresponding to different fault categories. This improvement demonstrates the model’s ability to learn and extract highly discriminative features, effectively distinguishing between various fault types in the feature space.

The experimental results comprehensively demonstrate the FCRNet model’s effectiveness in fault diagnosis. The high classification accuracy across different fault types, coupled with excellent performance metrics and clear feature separation, validates the model’s robust feature extraction and classification capabilities. The consistent performance across training, validation, and testing phases indicates strong generalization ability, making the proposed method reliable for practical fault diagnosis applications.

### 4.3 Comparative experiment

**Fig 16:**
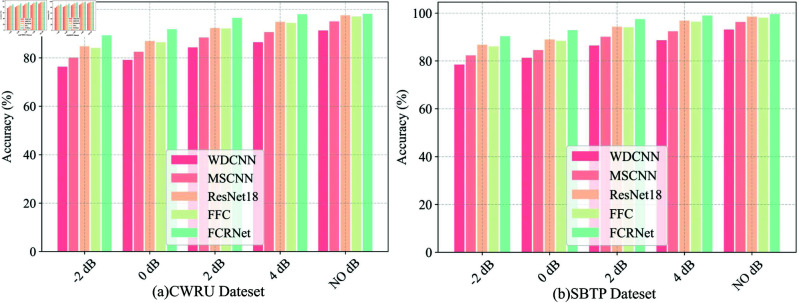
The accuracy of CWRU and SBTP dataset under different SNR.

Table 4 presents a comprehensive comparison of the proposed FCRNet with four state-of-the-art methods (WDCNN, MSCNN, ResNet18, and FFC) under various signal-to-noise ratio (SNR) conditions. The experimental results demonstrate the superior performance and robust noise immunity of FCRNet across all test scenarios. Fig 16 clearly illustrates the differences between the diagnostic algorithms and the variations in diagnostic performance of the same algorithm under different noise conditions.

**Table 4:** Performance comparison of different models in different SNR levels.

CWRU Dataset										
Algorithm	SNR = -2 dB		SNR = 0 dB		SNR = 2 dB		SNR = 4 dB		NO Noise	
	Accuracy%	F1%	Accuracy%	F1%	Accuracy%	F1%	Accuracy%	F1%	Accuracy%	F1%
WDCNN	76.34	75.89	79.12	78.45	84.32	83.87	86.45	85.98	91.34	90.56
MSCNN	80.12	79.45	82.54	81.92	88.43	87.98	90.67	90.21	95.02	94.87
ResNet18	84.78	84.12	86.98	86.45	92.34	91.87	94.89	94.21	97.56	97.45
FFC	84.12	83.87	86.45	85.89	92.12	91.56	94.45	94.01	97.12	97.01
FCRNet	**89.34**	**88.98**	**91.87**	**91.43**	**96.54**	**96.12**	**98.02**	**98.01**	**98.21**	**98.22**
SBTP Dataset										
Algorithm	SNR = -2 dB		SNR = 0 dB		SNR = 2 dB		SNR = 4 dB		NO Noise	
	Accuracy%	F1%	Accuracy%	F1%	Accuracy%	F1%	Accuracy%	F1%	Accuracy%	F1%
WDCNN	78.45	77.89	81.34	80.78	86.45	85.98	88.67	88.21	93.12	92.87
MSCNN	82.34	81.78	84.56	83.98	90.12	89.67	92.45	92.01	96.34	96.12
ResNet18	86.78	86.12	88.98	88.45	94.34	93.87	96.89	96.21	98.56	98.45
FFC	86.12	85.87	88.45	87.89	94.12	93.56	96.45	96.01	98.12	98.01
FCRNet	**90.34**	**89.98**	**92.87**	**92.43**	**97.54**	**97.12**	**99.02**	**99.01**	**99.62**	**99.63**

The comprehensive analysis of the performance comparison across different models and datasets reveals that FCRNet consistently outperforms other state-of-the-art methods under both noise-free and noisy conditions. In the CWRU dataset, FCRNet achieves the highest performance in the noise-free condition with 98.21% accuracy and 98.22% F1-score, marginally surpassing ResNet18 (97.56% accuracy) and FFC (97.12% accuracy). Notably, FCRNet maintains its superior performance even under increasingly challenging noise conditions. At SNR = 4 dB, FCRNet retains excellent performance with 98.02% accuracy, while other methods exhibit noticeable degradation. Even under severe noise conditions (SNR = -2 dB), FCRNet achieves 89.34% accuracy and 88.98 F1-score, substantially outperforming traditional methods like WDCNN (76.34% accuracy) and MSCNN (80.12% accuracy).

Similarly, in the SBTP dataset, FCRNet demonstrates robust performance across all SNR levels. In the absence of noise, FCRNet attains an impressive 99.62% accuracy and 99.63% F1-score, again slightly outperforming ResNet18 (98.56% accuracy) and FFC (98.12% accuracy). Under moderate noise conditions (SNR = 4 dB), FCRNet maintains a high accuracy of 99.02% and an F1-score of 99.01%, showcasing its resilience to noise. Even in the most adverse noise scenario (SNR = -2 dB), FCRNet achieves 90.34% accuracy and 89.98% F1-score, significantly exceeding the performance of WDCNN (78.45% accuracy) and MSCNN (82.34% accuracy).

Overall, the performance gap between FCRNet and other methods becomes more pronounced as noise levels increase, highlighting its robust feature extraction capability in adverse conditions. This superior noise immunity can be attributed to the effective integration of residual learning and Fast Fourier Convolution, which enables comprehensive feature extraction in both spatial and frequency domains. The consistent performance advantage across different noise levels demonstrates the practical applicability of FCRNet in real-world industrial environments where signal noise is inevitable.

### 4.4 Ablation experiment

**Fig 17:**
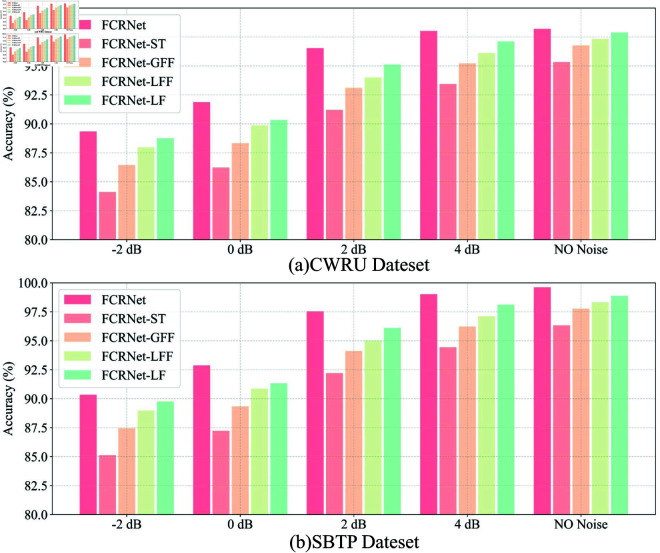
The accuracy of ablation experiment of CWRU and SBTP Dataset.

In the proposed FCRNet, the ST module is crucial, as its core function is to enhance feature representation through frequency domain analysis using GFF and LFF, addressing the limitations of traditional time-domain convolutional networks (LF operations) in capturing periodic or frequency information of vibration signals. By employing spectral transformations of local and global features, along with dimensionality reduction and filtering, it provides a richer and more robust feature representation. Therefore, this section conducts ablation experiments to investigate the impact of FCRNet’s internal structure on performance. In the experiments, the ST, GFF, LFF, and LF components of FCRNet are individually removed, and the model’s performance is tested under varying signal-to-noise ratios after each removal. Table 5 presents the specific numerical results of all tests, while Fig 17 illustrates the distribution and variation differences of the ablation experiments under different SNRs.

**Table 5:** Ablation experiment results of different feature fusions under different SNR.

CWRU Dataset										
Algorithm	SNR = -2 dB		SNR = 0 dB		SNR = 2 dB		SNR = 4 dB		NO Noise	
	Accuracy%	F1%	Accuracy%	F1%	Accuracy%	F1%	Accuracy%	F1%	Accuracy%	F1%
FCRNet	**89.34**	**88.98**	**91.87**	**91.43**	**96.54**	**96.12**	**98.02**	**98.01**	**98.21**	**98.22**
FCRNet-ST	84.12	83.89	86.23	85.92	91.21	90.89	93.45	93.12	95.34	95.12
FCRNet-GFF	86.45	86.12	88.34	87.98	93.12	92.76	95.23	94.87	96.78	96.56
FCRNet-LFF	87.98	87.65	89.87	89.54	94.01	93.67	96.12	95.78	97.34	97.12
FCRNet-LF	88.76	88.43	90.34	90.12	95.12	94.78	97.12	96.89	97.89	97.67
SBTP Dataset										
Algorithm	SNR = -2 dB		SNR = 0 dB		SNR = 2 dB		SNR = 4 dB		NO Noise	
	Accuracy%	F1%	Accuracy%	F1%	Accuracy%	F1%	Accuracy%	F1%	Accuracy%	F1%
FCRNet	**90.34**	**89.98**	**92.87**	**92.43**	**97.54**	**97.12**	**99.02**	**99.01**	**99.62**	**99.63**
FCRNet-ST	85.12	84.89	87.23	86.92	92.21	91.89	94.45	94.12	96.34	96.12
FCRNet-GFF	87.45	87.12	89.34	88.98	94.12	93.76	96.23	95.87	97.78	97.56
FCRNet-LFF	88.98	88.65	90.87	90.54	95.01	94.67	97.12	96.78	98.34	98.12
FCRNet-LF	89.76	89.43	91.34	91.12	96.12	95.78	98.12	97.89	98.89	98.67

The results of the ablation experiments indicate that the removal of any component leads to a noticeable decline in performance, underscoring the importance of each element in the architecture. Notably, the absence of the ST module results in the most significant performance degradation, with accuracy and F1-scores dropping substantially across all SNR levels in both the CWRU and SBTP datasets. For instance, in the CWRU dataset at SNR = -2 dB, the accuracy of FCRNet-ST falls to 84.12%, compared to 89.34% for the complete FCRNet. This highlights the critical role of the ST module in providing a richer and more robust feature representation through spectral transformations. Similarly, the removal of GFF and LFF also leads to reduced performance, emphasizing their contributions to capturing global and local frequency information, respectively. The LF component, while less impactful than the frequency-based modules, still plays a role in maintaining performance, particularly in higher SNR conditions.

These comparisons underscore ST’s essential enhancement of robustness through frequency-domain features, with LFF showing a stronger effect in noise, while GFF and LF provide complementary benefits. Future work could explore adaptive weighting of GFF and LFF for optimized performance across SNR levels.

## 5 Conclusions

In this study, we propose FCRNet, a fault diagnosis framework specifically designed for mining ventilation fan bearings, synergistically integrating residual learning and Fast Fourier Convolution to extract comprehensive spatial and frequency features. Extensive experimental validation on the CWRU and SBTP datasets demonstrates FCRNet’s superior performance, achieving macro-averaged F1-scores of 98.28% and 99.62%, respectively, while maintaining robust noise immunity across varying SNR levels. Comparative analyses reveal FCRNet consistently outperforms state-of-the-art methods, particularly in challenging noise conditions, attributable to its effective fusion of spatial and frequency-domain features. Ablation studies further confirm the pivotal contribution of the Spectral Transform module, alongside global and local frequency feature extraction mechanisms, in substantially enhancing diagnostic accuracy and robustness. These findings underscore FCRNet’s significant potential as a practical, sensor-driven diagnostic framework, offering reliable fault detection capabilities that can markedly enhance the safety and operational efficiency of mine ventilation systems.

## References

[1] Chatterjee A, Zhang L, Xia X. Optimization of mine ventilation fan speeds according to ventilation on demand and time of use tariff. Appl Energy. 2015;146:65–73. doi: 10.1016/j.apenergy.2015.01.134

[2] China Coal News. Establishing underground ventilation safety guardians to ensure smoother “breathing” for coal mines. 2024. https://www.chinacaj.net/i.16.93543.0.html

[3] Verified Market Reports. Market insights into underground mining ventilation systems. 2025. https://www.verifiedmarketreports.com/zh/product/underground-mining-ventilation-systems-market/

[4] El-Nagdy K. Stability of multiple fans in mine ventilation networks. Int J Mining Sci Technol. 2013;23(4):569–71.

[5] De Souza E. Application of ventilation management programs for improved mine safety. Int J Mining Sci Technol. 2017;27(4):647–50.

[6] Gou Y, Shi X, Zhou J, Qiu X, Chen X. Characterization and effects of the shock losses in a parallel fan station in the underground mine. Energies. 2017;10(6):785. doi: 10.3390/en10060785

[7] Martynenko V. Analysis of strength and bearing capacity of the auxiliary mine ventilation fan connected to the rotor of its electrical drive. In: 2020 IEEE KhPI Week on Advanced Technology (KhPIWeek). 2020. p. 19–23.

[8] Kursunoglu N, Onder M. Selection of an appropriate fan for an underground coal mine using the Analytic Hierarchy Process. Tunnel Undergr Space Technol. 2015;48:101–9.

[9] Wang D, Liu J, Lijun D, Honglin W. A supervised diagnostic experiment of resistance variable multifault locations in a mine ventilation system. Sci Rep. 2023;13(1):5259. doi: 10.1038/s41598-023-32530-7 37002333 PMC10066344

[10] Liu L, Liu J, Zhou Q, Huang D. Machine learning algorithm selection for windage alteration fault diagnosis of mine ventilation system. Adv Eng Inform. 2022;53:101666.

[11] Shen Z, Wang Q. Fault diagnosis of the mine ventilation system based on OCKIELM. AIP Advances. 2025;15(2).

[12] Yu S, Rong X, Wei L, Shi X. Review of fault diagnosis and early warning of coal mine ventilator. In: 2019 Chinese Automation Congress (CAC). IEEE; 2019. p. 5226–30.

[13] Cui W, Ding J, Meng G, Lv Z, Feng Y, Wang A, et al. Fault diagnosis of rolling bearings in primary mine fans under sample imbalance conditions. Entropy (Basel). 2023;25(8):1233. doi: 10.3390/e25081233 37628263 PMC10452977

[14] Zhang Q, Deng L. An intelligent fault diagnosis method of rolling bearings based on short-time Fourier transform and convolutional neural network. J Failure Anal Prevent. 2023;23(2):795–811.

[15] Kankar PK, Sharma SC, Harsha SP. Rolling element bearing fault diagnosis using wavelet transform. Neurocomputing. 2011;74(10):1638–45.

[16] Rai A, Upadhyay SH. A review on signal processing techniques utilized in the fault diagnosis of rolling element bearings. Tribol Int. 2016;96:289–306.

[17] Zhang S, Zhang S, Wang B, Habetler TG. Deep learning algorithms for bearing fault diagnostics—A comprehensive review. IEEE Access. 2020;8:29857–81.

[18] Li C, Li S, Feng Y, Gryllias K, Gu F, Pecht M. Small data challenges for intelligent prognostics and health management: a review. Artif Intell Rev. 2024;57(8):214.

[19] He W, Mao J, Li Z, Wang Y, Fang Q, Wu H. Fault identification of rotating machinery based on dynamic feature reconstruction signal graph. IEEE/ASME Trans Mechatron. 2023;29(3):2056–66.

[20] Wang D, Guo Q, Song Y, Gao S, Li Y. Application of multiscale learning neural network based on CNN in bearing fault diagnosis. J Signal Process Syst. 2019;91(10):1205–17.

[21] Ruan D, Wang J, Yan J, Gühmann C. CNN parameter design based on fault signal analysis and its application in bearing fault diagnosis. Advanced Engineering Informatics. 2023;55:101877.

[22] He W, Mao J, Wang Y, Li Z, Xie H, Shao H. Unified diagnostic and matching framework of fault and quality for robotic grinding system. IEEE Trans Instrument Measur. 2024.

[23] Guo Z, Yang M, Huang X. Bearing fault diagnosis based on speed signal and CNN model. Energy Rep. 2022;8:904–13.

[24] He W, Mao J, Wang Y, Li Z, Wang X, Wang S. Mqkin: Manufacturing quality knowledge-driven interpretable fault diagnosis network for robotic grinding equipment. IEEE Trans Automat Sci Eng. 2024.

[25] Jia L, Chow TW, Yuan Y. GTFE-Net: a gramian time frequency enhancement CNN for bearing fault diagnosis. Eng Appl Artif Intell. 2023;119:105794.

[26] Li C, Xie W, Zheng B, Yi Q, Yang L, Hu B. An enhanced CLKAN-RF framework for robust anomaly detection in unmanned aerial vehicle sensor data. Knowl-Based Syst. 2025:113690.

[27] Zhao B, Zhang X, Li H, Yang Z. Intelligent fault diagnosis of rolling bearings based on normalized CNN considering data imbalance and variable working conditions. Knowl-Based Syst. 2020;199:105971.

[28] Wang H, Sun W, He L, Zhou J. Rolling bearing fault diagnosis using multi-sensor data fusion based on 1D-CNN model. Entropy (Basel). 2022;24(5):573. doi: 10.3390/e24050573 35626458 PMC9141983

[29] Chen J, Jiang J, Guo X, Tan L. A self-adaptive CNN with PSO for bearing fault diagnosis. Syst Sci Control Eng. 2021;9(1):11–22.

[30] Wang H, Li C, Ding P, Li S, Li T, Liu C. A novel transformer-based few-shot learning method for intelligent fault diagnosis with noisy labels under varying working conditions. Reliab Eng Syst Safety. 2024;251:110400.

[31] Wang H, Liu Z, Peng D, Cheng Z. Attention-guided joint learning CNN with noise robustness for bearing fault diagnosis and vibration signal denoising. ISA Trans. 2022;128(Pt B):470–84. doi: 10.1016/j.isatra.2021.11.028 34961609

[32] Wu G, Ji X, Yang G, Jia Y, Cao C. Signal-to-image: rolling bearing fault diagnosis using ResNet family deep-learning models. Processes. 2023;11(5):1527.

[33] Peng D, Liu Z, Wang H, Qin Y, Jia L. A novel deeper one-dimensional CNN with residual learning for fault diagnosis of wheelset bearings in high-speed trains. IEEE Access. 2019;7:10278–93. doi: 10.1109/access.2018.2888842

[34] Wen L, Li X, Gao L. A transfer convolutional neural network for fault diagnosis based on ResNet-50. Neural Comput Appl. 2020;32(10):6111–24.

[35] Liang P, Wang W, Yuan X, Liu S, Zhang L, Cheng Y. Intelligent fault diagnosis of rolling bearing based on wavelet transform and improved ResNet under noisy labels and environment. Eng Appl Artif Intell. 2022;115:105269.

[36] Wallace K, Prosser B, Stinnette JD. The practice of mine ventilation engineering. Int J Mining Sci Technol. 2015;25(2):165–9.

[37] Grodzicka A, Plewa F, Krause M, Figiel A, Rozmus M. Selection of employees for performing work activities in currently used ventilation systems in hard coal mining. Energies. 2022;15(2):408.

[38] Yu H, Miao X, Wang H. Bearing fault reconstruction diagnosis method based on ResNet-152 with multi-scale stacked receptive field. Sensors (Basel). 2022;22(5):1705. doi: 10.3390/s22051705 35270851 PMC8915059

[39] Hou S, Lian A, Chu Y. Bearing fault diagnosis method using the joint feature extraction of transformer and ResNet. Measur Sci Technol. 2023;34(7):075108.

[40] Guan Q, Zhang Y, Yang W, Li X. Research on fault diagnosis method for fan rolling bearing based on improved ResNet50. In: 2024 2nd International Conference on Signal Processing and Intelligent Computing (SPIC). 2024. p. 779–83.

